# Discordance of light chain isotypes between serum and glomerular deposits in proliferative glomerulonephritis with monoclonal IgG deposits: a case report and review of the literature

**DOI:** 10.1186/s12882-023-03256-5

**Published:** 2023-07-01

**Authors:** Shoko Miura, Kan Katayama, Yuka Sugimoto, Fumika Tanaka, Mutsuki Mori, Daisuke Takahashi, Ryosuke Saiki, Yosuke Hirabayashi, Tomohiro Murata, Isao Tawara, Kaoru Dohi

**Affiliations:** 1 Department of Cardiology and Nephrology, Mie University Graduate School of Medicine, 2-174 Edobashi, Tsu, Mie 514-8507 Japan; 2 Department of Hematology and Oncology, Mie University Graduate School of Medicine, Tsu, Japan

**Keywords:** Discordance, Discrepancy, Light chain, Membranous nephropathy, Proliferative glomerulonephritis with monoclonal IgG deposits, Proteinuria

## Abstract

**Background:**

Proliferative glomerulonephritis with monoclonal immunoglobulin G (IgG) deposits (PGNMID) is a disease entity with nonorganized granular glomerular deposition with monoclonal proteins of both heavy and light chains. Dysproteinemia was observed in only 30% of the patients with PGNMID. We herein report a case of PGNMID with discrepancy between serum and glomerular deposits.

**Case presentation:**

The patient was a 50-year-old man who had been followed at a local clinic due to hypertension, type 2 diabetes, hyperlipidemia, hyperuricemia, fatty liver, and obesity. Proteinuria had been noted five years previously, and he had been referred to a hematology department due to hyperproteinemia, high gamma globulin, and κ Bence-Jones protein (BJP) positivity one year previously. Bone marrow aspiration showed 5% plasma cells, and he was referred to the nephrology department to evaluate persistent proteinuria. He was hypertensive, and his estimated glomerular filtration rate was 54.2 ml/min/1.73 m^2^. His urinary protein level was 0.84 g/g⋅Cr. Urine and serum immunofixation showed BJP-κ type and IgG-κ type, respectively. Kidney biopsy showed an increase in mesangial cells and matrix without nodular lesions under a light microscope. Immunofluorescence microscopy showed granular deposits of IgG and C3 on the capillary wall and weak positivity for C1q. IgG3 was predominant among the IgG subclasses, and intraglomerular κ and λ staining was negative for κ and positive for λ. Direct fast scarlet staining was negative. Electron microscopy showed lumpy deposits without a fibrillar structure in the subepithelial area. Based on the above findings, a diagnosis of membranous nephropathy-type PGNMID was made. Since proteinuria increased gradually after three years of treatment with valsartan (40 mg, daily), oral prednisolone (30 mg, daily) was initiated, which led to decreased proteinuria. The dose of oral prednisolone was gradually tapered to 10 mg per day. At that time, proteinuria was 0.88 g/g⋅Cr. We found 204 cases in 81 articles in the PubMed database, among which 8 showed discrepancy in the heavy and/or light chains between serum and kidney.

**Conclusions:**

We experienced a case of membranous nephropathy-type PGNMID with discrepancy in light chains between serum and kidney that was successfully treated with oral prednisolone.

## Background

Proliferative glomerulonephritis with monoclonal immunoglobulin G (IgG) deposits (PGNMID) was first reported as a disease entity with nonorganized granular glomerular deposition with monoclonal proteins of both heavy and light chains other than type 1 cryoglobulinemic glomerulonephritis, Randall type light and heavy chain deposition disease, immunotactoid glomerulonephritis, and fibrillary glomerulonephritis [1].

Dysproteinemia was observed in only 30% of the patients with PGNMID, and glomerular deposition with IgG3 was the most common finding [2]. After recognizing this disease entity, cases accumulated, some of which showed discrepancy between serum and glomerular deposits [3–8]. We herein report an additional case of PGNMID with discrepancy between serum and glomerular deposits.

## Case presentation

The patient was a 50-year-old man who had been followed up at a local clinic due to hypertension, type 2 diabetes, hyperlipidemia, hyperuricemia, fatty liver, and obesity. His medications included valsartan, voglibose, bezafibrate, and febuxostat. Although proteinuria was noted at an annual health checkup five years previously, proteinuria was not detected at the subsequent health checkup. Then, hyperproteinemia (9 g/dl), a high gamma globulin level, and κ Bence-Jones protein (BJP) positivity were noted, and he was referred to the hematology department of Mie University Hospital one year previously. Bone marrow aspiration showed 5% plasma cells, which did not lead to a diagnosis of multiple myeloma. Since he had persistent proteinuria, he was referred to the nephrology department and admitted for close examination.

His height was 170 cm, his body weight was 97.5 kg, and his body mass index was 33.7. His blood pressure was 151/103 mmHg. Although a physical examination did not reveal any obvious enlarged lymph nodes or hepatosplenomegaly, computed tomography (CT) showed mildly enlarged cervical, supraclavicular, axillary, mediastinal, para-aorta, hepatic portal, and bilateral inguinal lymph nodes, splenomegaly, and fatty liver. Positron emission tomography (PET)-CT showed mild accumulation of fluorodeoxyglucose in the cervical, supraclavicular, axillary, and inguinal lymph nodes. His laboratory data are shown in Table 1. His urinary protein level was 0.84 g/g⋅Cr without occult blood, and urine immunofixation showed BJP-κ type. Serum immunofixation showed IgG-κ type, and the blood immunoglobulin free light chain κ/λ ratio was also elevated (Table 1).

**Table 1:** Laboratory data

Urinary examination		Blood chemistry	
pH (4.5–7.5)	5.5	HbA1c (%, 4.9-6.0)	6.3
Protein (g/g⋅Cr)	0.84	Glu (mg/dl, 73–109)	154
Occult blood	-	TP (g/dl, 6.6–8.1)	9
Glucose	-	Alb (g/dl, 4.1–5.1)	4.4
β_2_mg (µg/l, 5-253)	< 30	BUN (mg/dl, 8–20)	15.9
NAG (U/l, 1.0-4.2)	11.4	Cr (mg/dl, 0.65–1.07)	0.98
		eGFR (ml/min/1.73m^2^)	54.2
Complete blood count		UA (mg/dl, 3.7–7.8)	6
WBC (/µl, 3300–8600)	4850	Na (mEq/l, 138–145)	141
RBC (×10^4^/µl, 435–555)	474	K (mEq/l, 3.6–4.8)	4.3
Hb (g/dl, 13.7–16.8)	14.6	Cl (mEq/l, 101–108)	106
Ht (%, 40.7–50.1)	41.8	Ca (mg/dl, 8.8–10.1)	9.4
Plt (×10^4^/µl, 15.8–34.8)	18.6	IP (mg/dl, 2.7–4.6)	3
		AST (U/l, 13–30)	53
Serology		ALT (U/l, 10–42)	48
ANA	2560	LDH (U/l, 124–222)	127
MPO-ANCA (U/ml, < 3.5)	< 0.5	ALP (U/l, 106–322)	175
Anti-SS-A (U/ml, 0–7.0)	240	γGTP (U/l, 13–64)	192
Anti-SS-B (U/ml, 0–7.0)	2.7	T-Bil (mg/dl, 0.4–1.5)	0.6
Anti-Sm (U/ml, < 7)	0.9	T-Chol (mg/dl, 142–248)	145
Anti-ds-DNA (U/ml, < 10)	4.5	TG (mg/dl, 40–234)	212
Anti-ss-DNA (U/ml, < 7)	16	CRP (mg/dl, 0-0.14)	0.09
C3 (mg/dl, 73–138)	92	IgG (mg/dl, 861–1747)	2767
C4 (mg/dl, 11–31)	6.1	IgG1(mg/dl, 351–962)	1920
CH50 (U/ml, 31.6–57.6)	43.5	IgG2(mg/dl, 239–838)	755
FLC κ (mg/l, 3.3–19.4)	213	IgG3(mg/dl, 8.5–140)	83.4
FLC λ (mg/l, 5.7–26.3)	74.2	IgG4(mg/dl, 4.5–117)	3.4
FLC κ/λ (0.26–1.65)	2.87	IgA (mg/dl, 93–393)	351
uIFE	BJP-κ	IgM (mg/dl, 33–183)	79
sIFE	IgG-κ	IgD (mg/dl, 0-12.6)	4.4
Cryoglobulin	-	β_2_mg (mg/l, < 2.0)	3.9

*Alb* Albumin, *ALP* Alkaline phosphatase, *ALT* Alanine transaminase, *ANA* Antinuclear antibody, *Anti-ds-DNA* Anti-double stranded DNA antibody, *Anti-Sm* Anti-Smith antibody, *Anti-SS-A* Anti–Sjögren’s-syndrome-related antigen A antibody, *Anti-ss-DNA* Anti-single stranded DNA antibody, *AST* Aspartate transaminase, *β2 mg* β2-microglobulin, *BJP* Bence Jones protein, *BUN* Blood urea nitrogen, *C3* Complement component 3, *C4* Complement component 4, *Ca* Calcium, *CH50* 50% hemolytic complement, *Cl* Chloride, *Cr* Creatinine, *CRP* C-reactive protein, *eGFR* Estimated glomerular filtration rate, *FLC* Free light chain, *γGTP* γ-glutamyltransferase, *Glu* Glucose, *Hb* Hemoglobin, *HbA1c* Hemoglobin A1c, *Ht* Hematocrit, *IgA* Immunoglobulin A, *IgD* Immunoglobulin D, *IgG* Immunoglobulin G, *IgM* Immunoglobulin M, *IP* Inorganic phosphates, *K* Kalium, *LDH* Lactate dehydrogenase, *MPO-ANCA* Myeloperoxidase anti-neutrophil cytoplasmic antibody, *Na* sodium, *NAG* N-acetyl-β-D-glucosaminidase, *Plt* Platelets, *RBC* Red blood cells, *sIFE* serum immunofixation, *T-Bil* Total bilirubin, *T-Chol* Total cholesterol, *TG* Triglyceride, *TP* Total protein, *UA* Uric acid, *uIFE* Urine immunofixation, *WBC* White blood cells

**Fig. 1:**
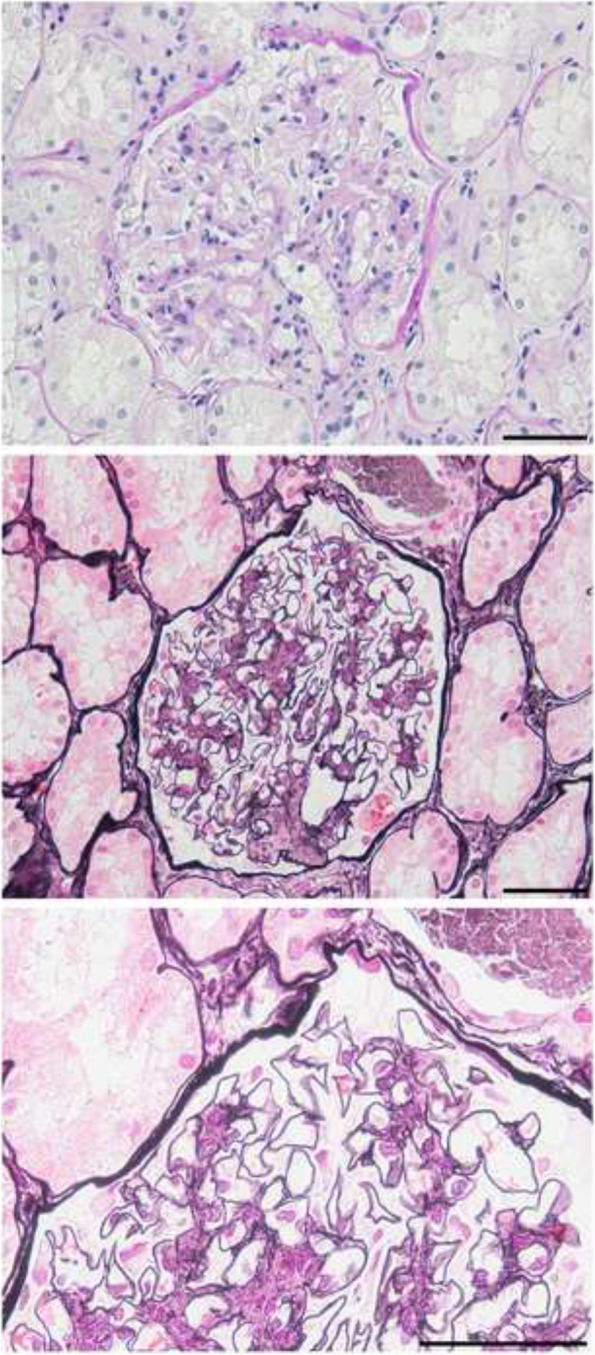
Light microscopy findings. There was an increase in mesangial cells and matrix without nodular lesions in periodic acid-Schiff staining (upper panel) or periodic acid-methenamine silver (PAM) staining (middle panel). There was no obvious spike formation under high magnification with PAM staining (lower panel). Bars = 50 μm

**Fig. 2:**
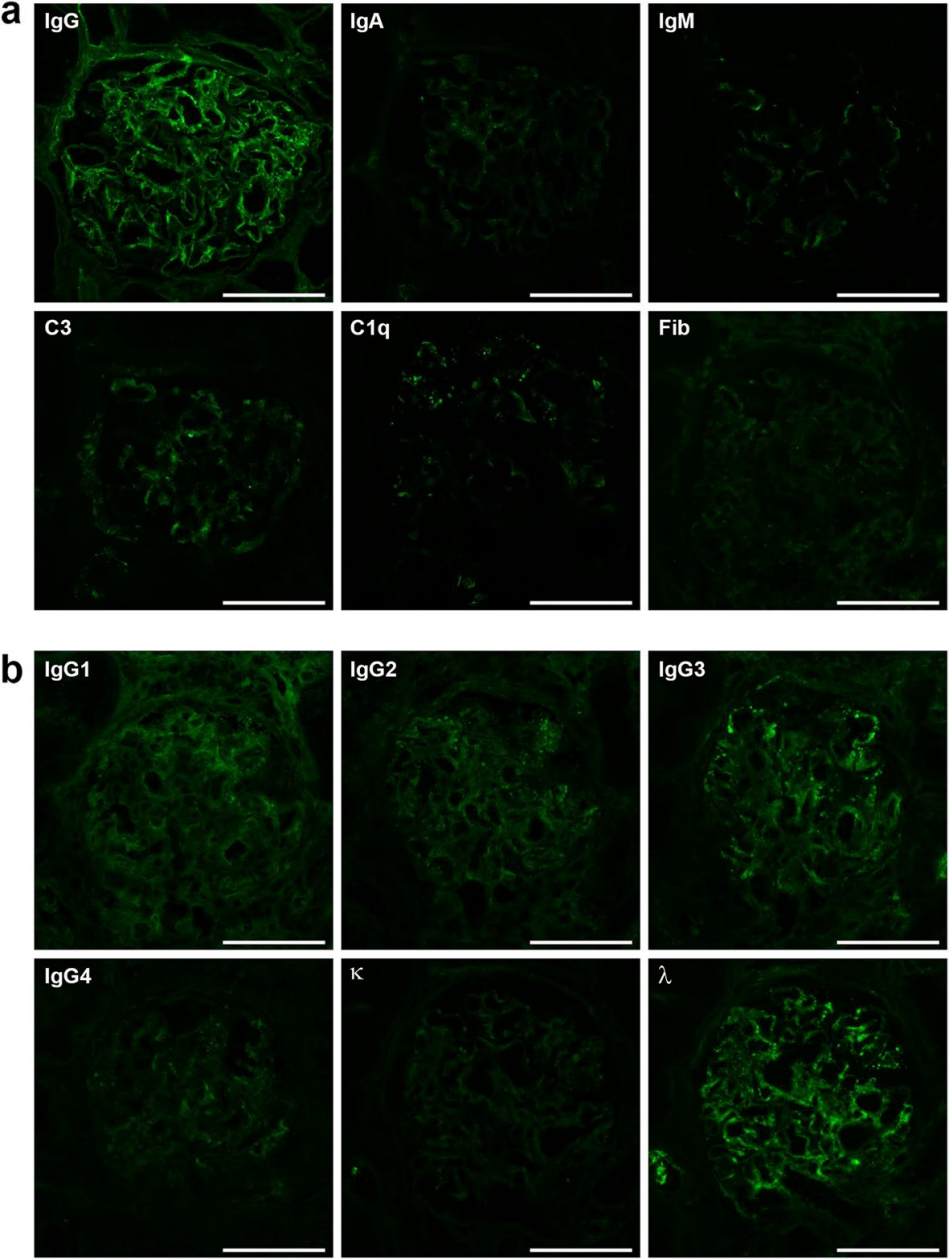
**a** Immunofluorescence findings. The immunofluorescence study showed granular deposits of immunoglobulin G (IgG) and complement 3 on the capillary wall and weak positivity for complement 1q. **b** IgG3 was predominant among the IgG subclasses, and intraglomerular κ and λ staining were negative for κ and positive for λ. Bars = 100 μm

**Fig. 3:**
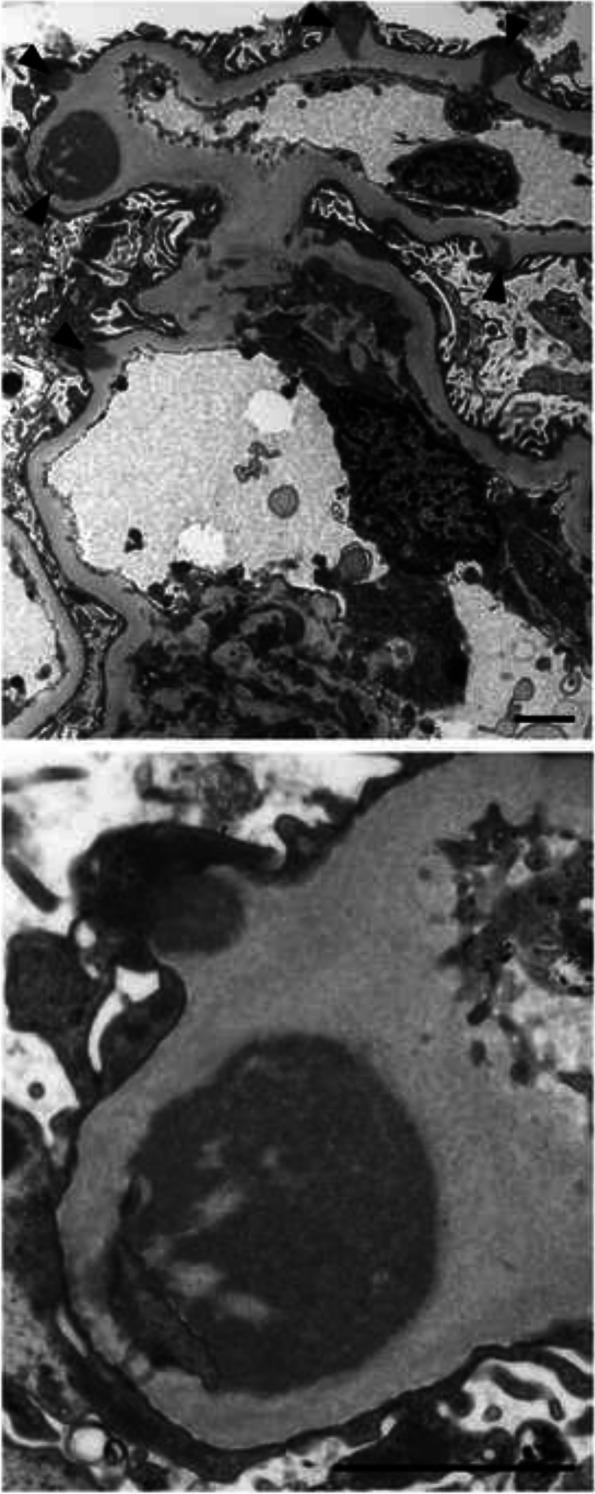
Electron microscopy findings. Electron microscopy showed electron-dense deposits (EDD) in the subepithelial area (arrowheads, upper panel). High magnification of the EDD showed granular nonorganized deposits without a fibrillar structure (lower panel). Bars = 2 μm

Kidney biopsy showed 24 glomeruli, of which 2 showed global sclerosis, 2 showed segmental sclerosis, and 1 collapsed. Tubulointerstitial damage was observed in 10–20% of patients. There was an increase in mesangial cells and matrix without nodular lesions under light microscopy (Fig. 1). No obvious spike formation was observed under high magnification with periodic acid methenamine silver staining (Fig. 1). An immunofluorescence study showed granular deposits of IgG and C3 on the capillary wall and weak positivity for C1q (Fig. 2a). IgG3 was predominant among the IgG subclasses, and intraglomerular κ and λ staining was negative for κ and positive for λ (Fig. 2b). Direct fast scarlet staining was negative. Electron microscopy revealed lumpy deposits without a fibrillar structure in the subepithelial area (Fig. 3).

**Fig. 4:**
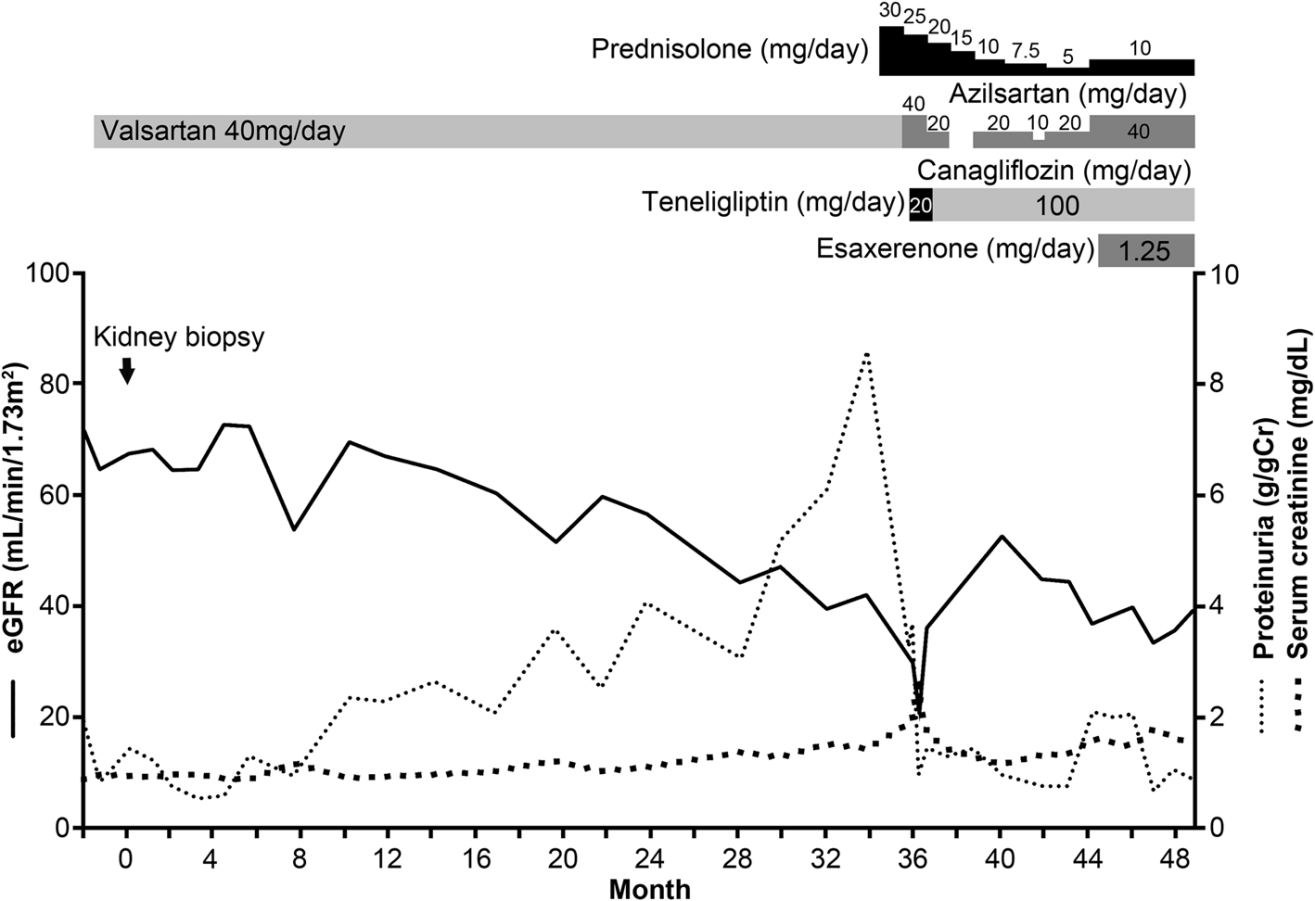
The clinical course. eGFR, estimated glomerular filtration rate

Based on the above findings, a diagnosis of membranous nephropathy-type PGNMID was made. Considering that hypertension and obesity-related hyperfiltration may have contributed to the urinary protein, he was treated with sodium restriction and body weight loss in addition to valsartan (40 mg, daily) (Fig. 4). Since his proteinuria increased gradually over the three years, oral prednisolone (30 mg, daily) was initiated, which led to a decrease in proteinuria. Valsartan was changed to azilsartan (40 mg, daily), and teneligliptin (20 mg, daily) was initiated. Oral prednisolone was gradually tapered to a dose of 10 mg per day, and his proteinuria was 0.88 g/g⋅Cr with the use of azilsartan (40 mg daily, canagliflozin (100 mg, daily), and esaxerenone (1.25 mg, daily).

## Discussion and conclusions

We experienced a case of PGNMID that showed a discrepancy in light chains between serum and kidney. While the present case had IgG-κ and BJP-κ in serum and urine, a bone marrow examination did not lead to a diagnosis of multiple myeloma. Kidney biopsy showed increased mesangial cells and matrix on light microscopy, and an immunofluorescence study showed granular deposits of IgG3 and λ light chain on the capillary wall. Direct first scarlet staining was negative, and electron microscopy showed granular electron-dense deposits in the subepithelial area. Based on the above findings, a diagnosis of membranous nephropathy-type PGNMID was made.

While IgG-κ was identified in the serum of the present case and IgG1 was predominant in the serum, the glomerular deposition was composed of IgG3-λ. Therefore, we searched the PubMed database using the term “glomerulonephritis with monoclonal IgG deposits” or “proliferative glomerulonephritis with monoclonal immunoglobulin deposits”. Among 204 cases in 81 articles, 8 cases showed discrepancy in heavy and/or light chains between serum and kidney (Table 2) [3–8]. Glomerular deposition of IgG, IgA, and IgM was observed in 5, 3, and 1 cases, respectively. Discrepancy of the heavy chain was reported in 4 cases. Discrepancy of the light chain was reported in 7 cases. Regarding the histological pattern, mesangial proliferative glomerulonephritis, membranoproliferative glomerulonephritis, and membranous nephropathy were found in 4, 3, and 2 cases, respectively. Although the significance of discrepancy in κ and λ is not clear, IgG3 is characterized by a strong positive charge and large molecular weight, which might explain its affinity to the glomeruli [2]. In the present case, IgG1-κ was positive in serum, and IgG3-λ was positive in the glomeruli, suggesting that the affinity of IgG3-λ to the glomeruli caused a discrepancy between serum and kidney.

**Table 2:** Summary of reported cases of PGNMID with discrepancy between serum and glomerular deposits

	Article	Age	Sex	Cr (mg/dl)	eGFR (ml/min/1.73m^2^)	Proteinuria (g/g⋅Cr)	Glomerular deposition	FLC κ (mg/dl)	FLC λ (mg/dl)	Circulating paraprotein (method of detection)	Histological pattern
1	Redondo-Pachón 2012	76	M	3.4	18	11	IgG-λ	ND	ND	IgA-λ (sPEP) IgA-λ (uPEP)	MPGN
2	Batal 2014 pat 4	61	M	1.8	39	ND	IgG1-κ	ND	ND	IgM-λ (sIFE)	MPGN
3	Batal 2014 pat 7	44	F	ND	ND	ND	IgA-κ	ND	ND	IgG-κ (sIFE)	Mes GN
4	Vignon 2017 pat 1	ND	ND	ND	ND	ND	IgA-κ	ND	ND	IgA-λ (sIFE) λ (uIFE)	Mes GN
5	Vignon 2017 pat 5	ND	ND	ND	ND	ND	IgA-λ	35	36	IgA-κ (Western)	Mes GN
6	Shimohata 2017	76	M	3.6	17	4.9	IgG1-λ	ND	ND	IgG-κ (sPEP) IgG-κ (uPEP)	MN
7	Gumber 2018 pat 8	65	M	2	34	9.52	IgM-κ	ND	ND	IgG-κ IgG-λ (sPEP)	MPGN
8	Rosenstock 2021 pat 2	25	F	1.4	54	0.296	IgG1-κ	ND	ND	λ (sIFE)	Mes GN
9	The present case	50	M	0.98	54.3	0.84	IgG3-λ	213	74.2	IgG-κ (sIFE) BJP-κ (uIFE)	MN

*BJP* Bence Jones protein, *Cr* Creatinine, *eGFR* Estimated glomerular filtration rate, *F* Female, *FLC* Free light chain, *IgG* Immunoglobulin G, *M* Man, *ND* Mes GN, Mesangial proliferative glomerulonephritis, *MN* Membranous nephropathy, *MPGN* Membranoproliferative glomerulonephritis, no data, *pat* patient, *sIFE* Serum immunofixation, *sPEP* Serum protein electrophoresis, *uIFE* Urine immunofixation, *uPEP* Urine protein electrophoresis

Since there is no definitive treatment for PGNMID [9], renin–angiotensin system inhibitors were considered to be a reasonable treatment for the present case. Initially, the patient showed a decrease in urinary protein with valsartan alone; however, his proteinuria gradually increased, and kidney dysfunction also appeared. Since a previous report showed successful treatment with oral prednisolone in a case of membranous nephropathy-type PGNMID with IgG2-κ deposition [10], oral prednisolone was started, and proteinuria decreased in the present case. A clone-directed approach will be needed for cases with aggravation of kidney function and proteinuria in the future [7, 11].

The anti-nuclear antibody (ANA) and anti-SS-A antibody levels were high in this case (2560 times and 240 times, respectively). According to the 2019 European League Against Rheumatism (EULAR)/American College of Rheumatology (ACR) classification criteria for systemic lupus erythematosus (SLE) [12], the present case had urinary protein > 0.5 g/day (4 points) and low C4 (3 points) but no other systemic symptoms, blood findings, or abnormal findings in the skin, mucosa, or musculoskeletal system; thus, a diagnosis of SLE was not made. While the C4 level was low, we did not investigate the presence of immune complex in serum by a C1q binding assay or a monoclonal Rheumatoid factor assay in the present case. There were also no findings of dryness of the eyes and no abnormalities of the salivary or parotid glands; thus, a definitive diagnosis of Sjögren’s syndrome could not be made. However, hypergammaglobulinemia might imply the subclinical status of these autoimmune diseases, and there was a possibility of the contribution of polyclonal gammopathy to the discrepancy of this serum and glomerular light chain dominancy. As there was no description of the presence of hypergammaglobulinemia in the other eight cases in Table 2, we could not elucidate whether there was a common feature of hypergammaglobulinemia that was related to the discrepancy between serum and kidney. There might be a monoclonal gammopathy of undetermined significance with IgG1-κ, which did not contribute to kidney injury, and a monoclonal gammopathy of renal significance with IgG3-λ, which was specifically deposited in the kidney in the present case. The presence of two B-cell clones producing different M proteins was difficult to prove in the flow cytometry analysis of the bone marrow of the present case. While the majority were IgG1-κ-producing B-cell clones in the present case, a small amount of IgG3-λ B-cell clones were present, which could not be detected in serum or urine and which tended to be deposited in the kidney at the same time [13]. This is compatible with a previous study that reported that the rate of detectable monoclonal immunoglobulins in PGNMID was low at 30–32% [14].

The present case was diagnosed with membranous nephropathy-type PGNMID, which was almost synonymous with nonorganized and non-Randall-type monoclonal immunoglobulin deposition disease associated with membranous features. While there was a report of a case with membranous nephropathy with monoclonal IgM-λ deposition that showed THSD7A positivity [15], we could not search for antigens associated with membranous nephropathy (e.g., PLA2R, THSD7A, and NELL1) due to a lack of samples in the present case; therefore, it was impossible to distinguish the present case from primary membranous nephropathy with monoclonal immunoglobulin deposits.

In conclusion, we experienced a case of membranous nephropathy-type PGNMID with a discrepancy in light chains between serum and kidney.

## Data Availability

The datasets used and/or analyzed during the current study are available from the corresponding author on reasonable request.

## Abbreviations

ACR: American College of Rheumatology
ANA: Anti-nuclear antibodies
BJP: Bence-Jones protein
CT: Computed tomography
EULAR: European League Against Rheumatism
IgG: Immunoglobulin G
PET: Positron emission tomography
PGNMID: Proliferative glomerulonephritis with monoclonal IgG deposits
SLE: Systemic lupus erythematosus
